# Stuffy nose disorder: a new disorder stemming from prolonged hydroxychloroquine therapy

**DOI:** 10.1097/MS9.0000000000001947

**Published:** 2024-03-15

**Authors:** Ryan Varghese, Mainak Bardhan, Vinay Suresh, Tirth Dave

**Affiliations:** a Department of Pharmaceutical Sciences, Philadelphia College of Pharmacy, Philadelphia, PA; b Miami Cancer Institute, Baptist Health South Florida, Coral Gables, FL; c King George’s Medical University, Lucknow, India; d Bukovinian State Medical University, Chernivtsi, Ukraine

Originally indicated for the treatment of malaria, hydroxychloroquine (HCQ), a steroid-sparing anti-inflammatory agent, has been the cornerstone for the management of several rheumatic autoimmune diseases (RADs), including rheumatoid arthritis (RA), primary Sjögren’s syndrome (SS), and most commonly systemic lupus erythematosus (SLE)^1^. Owing to an established safety profile and cost-utility ratio, HCQ upholds its position as a disease-modifying anti-rheumatic drug (DMARD), despite having an ill-established mechanism of action^1^. Growing evidence suggests that HCQ manages the flare-up of these RADs by intercepting endolysosomal functions and autophagy, thereby affecting signalling pathways and gene transcription^2^. Ultimately, this leads to inhibition of the production of pro-inflammatory cytokines and other co-stimulatory chemokines^2^. The mechanism of action and the potential of HCQ in the clinical armamentarium against various RADs have been summarized by Danza *et al.* ^3^. Currently, HCQ is being explored for the management of dermatomyositis, sarcoidosis, and Still’s disease, to name a few^3^.

The most common adverse effects of chronic administration of HCQ include retinopathy, myopathy, and iatrogenic cardiomyopathy^2^. While there have been no reports of nasal issues associated with HCQ, recent evidence has raised concerns about the use of HCQ owing to a potential association between the prolonged administration of this drug and nasal side effects. We would propose the term ‘Stuffy Nose Disorder (SND)’ to encapsulate this phenomenon. This term signifies the nasal and nasopharyngeal side effects caused by prolonged HCQ therapy, irrespective of the indication. The term also emphasizes a novel framework for understanding the mechanisms behind SND and offers a promising approach for the detection and management of the same.

A phase-IV clinical study (*n* = 40 773 patients) of the Food and Drug Administration (FDA) data, revealed a potential link between hydroxychloroquine (HCQ) sulfate with nasal congestion and SND. Of the cohort, 235 (0.58%)^4^ reported SND following prolonged therapy. Primarily, this correlation was profound in women aged between 50 and 59 years, who had been on HCQ regimen for about 2–5 years^4^. This could be related to the sex bias in autoimmune diseases, wherein women account for 90% and 75% of the total cases of SLE and RA, respectively^5^. Furthermore, the age group reflected in the phase-IV clinical study could be attributed to the higher rates of RADs in perimenopausal (40–49 years) and post-menopausal (50–55 years) women^6^, who have been on prolonged (2–5 years)^4^ HCQ therapy. The diminished incidence of SND could potentially be attributed to the fact that only a fraction of the population was prescribed HCQ on a prolonged basis (2–5 years).

In addition, there have been only 28 cases reported between 2004 and 2018, and 207 cases between 2019 and 2022^4^, which could be potentially attributed to misinformation about the efficacy of HCQ in the management of SARS-CoV-2 symptoms^7^. While there have been no case reports published on this topic, the data could be supplemented by patient testimonials in rheumatology discussion forums^8^.

We believe that SND, similar to retinopathy, could be attributed to the characteristic volume of distribution (V_d_)^2^ and long half-life (t_50_) of HCQ, which results in prolonged effects even after discontinuation of therapy^2^. Given the growing prevalence of RADs^9^, the exact mechanisms underlying SND need to be elucidated comprehensively. In addition, dose reduction or temporary cessation of therapy could be considered upon the precipitation of SND-related symptoms. Nonetheless, a lacuna in the understanding of the dose–response relationships of HCQ and the lack of well-defined lower and upper dose limits for RADs pose a significant challenge^2^. Furthermore, the effect of concurrent administration of medications, including non-steroidal anti-inflammatory drugs (NSAIDs) prescribed during the perimenopausal and post-menopausal periods, needs to be investigated further.

In addition, given the aforementioned limitations, it is practically impossible to tailor doses to meet patient-specific needs. Thus, similar to HCQ-induced retinopathy, it is paramount to conduct regular tests to eliminate the risk of SND. Additionally, tests such as nasal endoscopy and computed tomography (CT) scans could prove beneficial for monitoring the severity and tracking the velocity of SND progression. Furthermore, similar to screening for retinopathy, it is advisable to consolidate the screening for SND on an annual basis for the initial two years of HCQ therapy. Subsequently, the screening could be segued to biannual examinations throughout the remainder of the therapeutic regimen. If SND is minor and does not affect the quality of life (QoL) of the patient, the dose of HCQ can be tapered or temporarily ceased without compromising disease management. However, in cases where the patient QoL is being affected by clinical manifestations such as dyspnoea, apnoea, and anosmia, the rheumatologists and otorhinolaryngologists should determine the best therapeutic or surgical intervention on a case-by-case basis. Switching to a different DMARD or prescribing a combination therapy that is well tolerated by the patient may help in retarding the progression of SND. In cases requiring hospital admission or immediate medical intervention, surgical modalities, such as paranasal sinus-functional endoscopic sinus surgery (PNS-FESS), can be explored, adhering to the preoperative CT evaluation protocol. Symptomatic treatments, such as antihistamines, decongestants, and saline nasal sprays, can be recommended to provide temporary relief to patients.

## Ethics approval

Not applicable.

## Consent

Informed consent was not required for this editorial.

## Source of funding

This research did not receive any specific grant from funding agencies in the public, commercial, or not-for-profit sectors.

## Author contribution

All author contributed equally in drafting the manuscript but R.V. and M.B. qualify for joint first authorship.

## Conflicts of interest disclosure

The authors declare that there are no competing interests.

## Research registration unique identifying number (UIN)

Not applicable.

## Guarantor

Ryan Varghese.

## Data availability statement

The data in this correspondence article are not sensitive in nature and are accessible in the public domain. The data are therefore available and not of a confidential nature.

## Provenance and peer review

Not commissioned, internally peer-reviewed.
